# First detection of *kdr* L1014F allele in *Anopheles ziemanni* and *Anopheles pharoensis* in Cameroon and distribution of the allele in members of the *Anopheles gambiae* complex

**DOI:** 10.1186/s13071-024-06420-4

**Published:** 2024-08-27

**Authors:** Marie Paul Audrey Mayi, Christophe Antonio-Nkondjio, Roland Bamou, Claudia Damiani, Alessia Cappelli, Borel Djiappi-Tchamen, Landre Djamouko-Djonkam, Mahdokht Ilbeigi Khamseh Nejad, Verena Pichler, Irene Ricci, Guido Favia

**Affiliations:** 1 School of Biosciences and Veterinary Medicine, University of Camerino, Via Gentile III da Varano, 62032 Camerino, Italy; 2 Institut de Recherche de Yaoundé (IRY), Organisation de Coordination pour la lutte contre les Endémies en Afrique Centrale (OCEAC), P.O. Box 288, Yaoundé, Cameroon; 3 Vector Biology, Liverpool School of Tropical Medicine, Pembroke Place, Liverpool, L3 5QA UK; 4 Vector Borne Diseases Laboratory of the Research Unit for Biology and Applied Ecology (VBID-RUBAE), Department of Animal Biology, Faculty of Science of the University of Dschang, Dschang, Cameroon; 5 Laboratory of Malaria and Vector Research-LMVR, Rockville National Institute of Health /NIAID, Rockville, USA; 6 School of Biosciences and Veterinary Medicine, University of Camerino, CIRM Italian Malaria Network, Via Gentile III da Varano, 62032 Camerino, Italy; 7 Department of Public Health and Infectious Diseases, Sapienza University of Rome, Rome, Italy

**Keywords:** *Anopheles gambiae*, *Anopheles coluzzii*, *Anopheles arabiensis*, *Anopheles pharoensis*, *Anopheles ziemanni*, Cameroon, Insecticide resistance, *kdr* mutation, Malaria

## Abstract

**Background:**

Knockdown resistance (*kdr*) is one of the primary resistance mechanisms present in anopheline species. Although this mutation is largely spread across the *Anopheles gambiae* s.l. members, its prevalence in other species is still not well documented.

**Methods:**

The present study investigated the distribution and allelic frequencies of *kdr* in *An. gambiae* s.l., *An. pharoensis*, and *An. ziemanni* samples collected in 2022 and 2023 in nine sites spread across five ecogeographical settings in Cameroon. Members of the *An. gambiae* complex were identified molecularly by polymerase chain reaction (PCR). *kdr* L1014F and L1014S alleles were screened by PCR and confirmed by sequencing.

**Results:**

*An. gambiae* (49.9%), *An. coluzzii* (36.5%), and *An. arabiensis* (13%) were identified, and the frequency of the *kdr* L1014F was high in both *An. gambiae* and *An. coluzzii* in all sites. The *kdr* L1014F allele was detected for the first time in 8 out of 14 *An. ziemanni* samples examined and in 5 out of 22 *An. pharoensis* samples examined. The *kdr* L1014S allele was scarce and found only in the heterozygote “RS” state in *An. arabiensis* and *An. gambiae* in Yangah and Santchou.

**Conclusions:**

The present study sheds light on the rapid expansion of the *kdr* L1014F allele in malaria vectors in Cameroon and stresses the need for surveillance activities also targeting secondary malaria vectors to improve the control of malaria transmission.

**Graphical Abstract:**

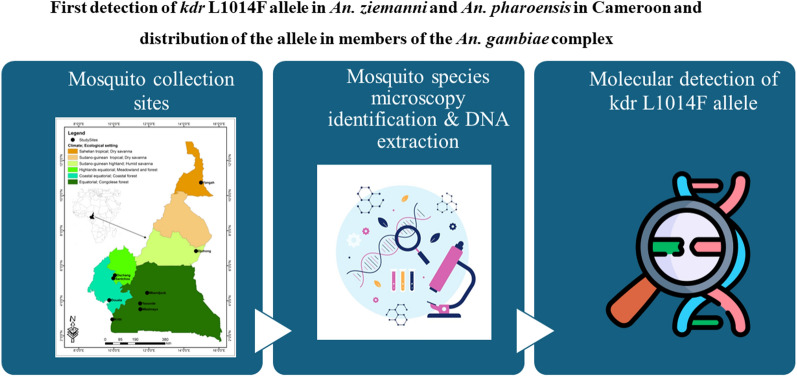

## Background

Malaria remains a major public health problem in Cameroon [1]. In 2022, there were over six million malaria cases reported in health care centers across the country. It is estimated that 24% of the 25 million Cameroonians have at least one malaria attack each year [2]. Disease incidence is estimated to vary between 100 and 196 per 1000 according to epidemiological records [1]. Despite the frequent distribution of bed nets across the country, there has not been a significant decline of malaria [1]. Among factors affecting vector control measure performance are the rapid expansion of insecticide resistance and the high diversity of vector populations, which display different feeding, resting, and biting behaviors [3]. Studies characterizing resistance mechanisms in vector populations indicated a rapid increase of insecticide resistance in *Anopheles gambiae* s.l. and *An. funestus* with multiple resistance profiles [4–7]. Recent studies also indicated a reduced level of insecticide susceptibility of several other anopheline species, including *An. moucheti*, *An. coluzzii*, *An. nili*, and *An. rufipes* to dichloro-diphenyl-trichloroethane (DDT) and pyrethroids [8–10]. Apart from *An. gambiae* s.l. and *An. funestus* for which resistance mechanisms have been extensively explored, few studies characterizing resistance mechanisms in other anopheline species have been undertaken [9].

Different mechanisms including metabolic, cuticular, and target site mutations [e.g., knockdown resistance (*kdr*)] drive resistance to insecticides in mosquitoes [11]. *kdr* mutations, among the most widely spread resistance mechanisms, consist in aminoacidic substitutions in the voltage-gated sodium channel (Vgsc) that reduce the binding and/or action of pyrethroids and DDT and, thus, result in a reduced susceptibility to these insecticides [12, 13]. This resistance mechanism is highly frequent in *An. gambiae* with two widespread resistance alleles: the L1014F allele widely distributed in West and Central Africa and the L1014S allele more frequent in Eastern Africa [14–16]. However, there are still not enough data on the distribution of these alleles in other *Anopheles* species. The present study investigated the distribution of these alleles in *An. gambiae*, *An. coluzzii*, *An. arabiensis*, *An. pharoensis*, and *An. ziemanni* mosquitoes collected across Cameroon.

**Fig. 1:**
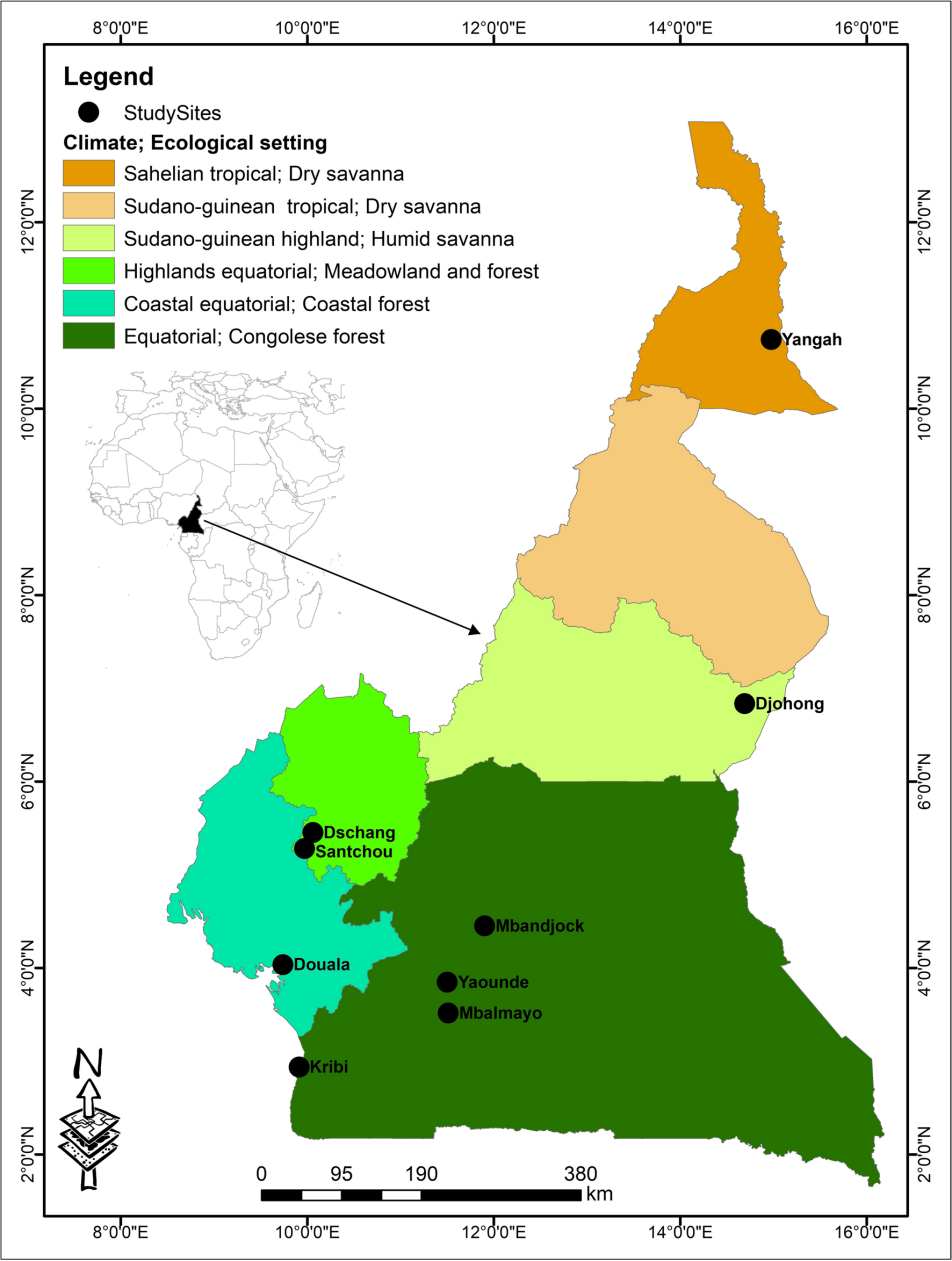
A map of Cameroon showing the collection sites. The nine collection sites (black dots) are distributed in five of the six ecogeographical areas

Mosquitoes were collected from nine locations belonging to five different ecogeographical areas in Cameroon (dry savanna, humid savanna, highlands, coastal, and forest) (Fig. 1 and Table 1) during the periods of September to November 2022 and June to August 2023 in the raining season, using different sampling methods, including Centers for Disease Control light traps, human landing catches, and Prokopack aspirators. Adult mosquitoes were identified morphologically using the identification keys of Gillies and Coetzee (1987) [17] and Gillies and De Meillon (1968) [18] and preserved in silica gel for molecular analyses.

**Table 1:** Descriptions of the study sites

Sites	Regions	Ecological settings	Climate	AAT (°C)	Seasons	Crops
Yangah	Far North	Dry savanna	Sahelian	33	4 months rainy/8 months dry	Cotton, rice, millet, sorghum, maize
Djohong	Adamawa	Humid savanna	Sudano-Guinean highland	24	7 months rainy/5 months dry	Cotton, coffee millet, sorghum
Dschang	West	Meadowland and forest	Highlands equatorial	21.6	8 months rainy/4 months dry	Coffee, Irish potatoes, maize, cabbage, taro
Santchou	West	Meadowland and forest	Highlands equatorial	22.5	8 months rainy/4 months dry	Maize, cassava, sweet potato, cocoyam, cocoa, coffee
Douala	Littoral	Coastal forest	Coastal equatorial	27.5	9 months rainy/3 months dry	Cocoa, oil palm, rubber, plantain, banana, cassava, yams
Yaoundé	Center	Congolese forest	Equatorial	24	9 months rainy/3 months dry	Cocoa, coffee, yams
Mbandjock	Center	Congolese forest	Equatorial	26.5	7 months rainy/5 months dry	Sugarcane, oil palm, maize, cassava, yams
Mbalmayo	Center	Congolese forest	Equatorial	25	9 months rainy/3 months dry	Cocoa, coffee, cassava, yams, maize
Kribi	South	Congolese forest	Equatorial	26	9 months rainy/3 months dry	Cocoa, oil palm, rubber, cassava, plantains, yams

*AAT *average annual temperature

DNA extraction was done with the JETFLEX Genomic DNA Purification Kit (Invitrogen by Thermo Fisher Scientific) following the manufacturer’s guidelines. Members of the *An. gambiae* complex were identified using the rapid high-throughput SYBR green assay described by Chabi et al. (2019) [19] and/or using the protocol of Favia et al. (2001) [20]. Some *An. phaorensis* and *An. ziemanni* samples were sequenced at cytochrome c oxidase subunit 1 (COI) loci for species confirmation [21].

A subset of mosquito species collected from each site were used for the screening of *kdr* alleles 1014L/S. Allele-specific polymerase chain reaction (AS-PCR) was used to detect L1014F (AS-PCR Agd3) and L1014S (AS-PCR Agd5) alleles as described by Verhaeghen et al. (2006) [22]. Some samples of *An. gambiae* s.l., *An. ziemanni*, and *An. pharoensis* were later Sanger sequenced for the confirmation of the presence/absence of the mutation at the Microsynth Company (Germany).

After checking the quality of the chromatograms, we blasted the sequences (https://blast.ncbi.nlm.nih.gov/Blast.cgi?PROGRAM=blastn&PAGE_TYPE=BlastSearch&LINK_LOC=blasthome) and aligned them in reverse and forward direction using ClustalW (https://www.genome.jp/tools-bin/clustalw). The *kdr* L1014F and L1014S mutations were detected studying the picks of the chromatograms corresponding to the mutation sites.

A total of 649 *Anopheles* mosquitoes (*An. gambiae* s.l. (*N* = 507), *An. pharoensis* (*N* = 48), and *An. ziemanni* (*N* = 94)) were collected and examined. *Anopheles gambiae* s.l. samples (Djohong *N* = 48, Douala *N* = 64, Mbalmayo *N* = 39, Mbandjock *N* = 41, Santchou *N* = 47, Yangah *N* = 82, Yaoundé *N* = 66, Kribi *N* = 58, Dschang *N* = 62) were screened molecularly to the species level. PCR results revealed three species belonging to the *An. gambiae* complex: *An. gambiae* (49.9%), *An. coluzzii* (36.5%), and *An. arabiensis* (13%). A few hybrids (*An. gambiae*/*An.coluzzii*) were also recorded (0.6%).

*Anopheles gambiae* was recorded in almost all sites, while *An. arabiensis* was only found in Yangah together with *An. pharoensis* and *An. ziemanni*. In Mbanjock, Djohong and Dschang, only *An. gambiae* was found, whereas in Kribi, only *An. coluzzi* was registered. Both *An. gambiae* and *An. coluzzii* were found in sympatry in Mbalmayo, Yaoundé, and Douala.

The *kdr* allele L1014F was found at very high frequency in both *An. gambiae* (PQ000897) and *An. coluzzii* (PQ000899) in all sites, while only 2 *An. arabiensis* out of 59 were found with the allele (PQ000905) (Table 2). The *kdr* allele L1014S was scarce and detected only at the heterozygote “RS” state in *An. arabiensis* (PQ000906) and *An. gambiae* in Yangah and Santchou. One *An. gambiae* sample (PQ000898) was found with the double mutation L1014F/S (Table 2). It is noteworthy that the *kdr* allele L1014F was detected for the first time in *An. ziemanni* and *An. pharoensis*: out of the 14 *An. ziemanni* examined, 7 were found to be homozygotes “RR” (PQ000903) and 1 was heterozygote “RS” (PQ000901). Out of the 22 *An. pharoensis* examined, three were found to be homozygotes “RR” (PQ000907) and 2 were heterozygote “RS” (PQ000909) (Table 2). No *kdr* 1014S was detected in *An. ziemanni* and *An. pharoensis*.

**Table 2:** Distribution of the *kdr* alleles L1014F/S in anopheline species collected in different sites across Cameroon

			L1014F				L1014S			
Sites	Species	N	RR (%)	RS (%)	SS (%)	Allele freq	RR (%)	RS (%)	SS (%)	Allele freq
Yangah	*An. coluzzii*	12	7(58.3)	5(41.7)	0(0)	0.79	0	0	12(100)	0
	*An. arabiensis*	59	0(0)	2(3.4)	57(96.6)	0.02	0	5(10)	45(90)	0.05
	*An. pharoensis*	22	3(13.6)	2(9.1)	17(77.3)	0.18	0	0	0	0
	*An. ziemanni*	14	7(50)	1(7.1)	6(42.9)	0.54	0	0	0	0
Djohong	*An. gambiae*	20	20(100)	0(0)	0(0)	1	0	0	20(100)	0
Santchou	*An. gambiae*	20	20(100)	0(0)	0(0)	1	0	1(5)	19(95)	0.03
Douala	*An. gambiae*	20	20(100)	0(0)	0(0)	1	0	0	20(100)	0
	*An. coluzzii*	20	14(70)	6(30)	0(0)	0.85	0	0	20(100)	0
Yaounde	*An. gambiae*	20	20(100)	0(0)	0(0)	1	0	0	20(100)	0
	*An. coluzzii*	20	17(85)	3(15)	0(0)	0.92	0	0	20(100)	0
Mbandjock	*An. gambiae*	20	20(100)	0(0)	0(0)	1	0	0	20(100)	0
Mbalmayo	*An. gambiae*	5	5(100)	0(0)	0(0)	1	0	0	5(100)	0
	*An. coluzzii*	20	16(80)	4(20)	0(0)	0.9	0	0	20(100)	0
Kribi	*An. coluzzii*	58	48(82.8)	10(17.2)	0(0)	0.91	0	0	58(100)	0
Dschang	*An. gambiae*	62	62(100)	0(0)	0(0)	1	0	0	62(100)	0

*N* sample size, *Allele freq* frequency of resistance allele

The present study objective was to investigate the presence of *kdr* alleles 1014F/S and their frequencies in mosquito samples collected from different parts of Cameroon. The study indicated a high prevalence of the *kdr* allele L1014F in both *An. gambiae* and *An. coluzzii* in all study sites. This result was similar to studies conducted so far in Cameroon reporting a high frequency of the *kdr* resistance allele in members of the *An. gambiae* complex [4, 23–25].

Interestingly, the allele L1014F was also detected for the first time in both *An. ziemanni* and *An. pharoensis*. These species are considered as secondary malaria vectors in Cameroon owing to their low implication in malaria transmission and their highly zoophilic and exophilic behavior [3, 26]. The presence of this mutation in these species could result from the high selective pressure induced using pesticides in agriculture. Indeed, the site of Yangah where *An. ziemanni* and *An. pharoensis* were sampled is a locality where rice, millet, and cotton are cultivated in large surfaces. The production of these crops requires the use of large quantities of pesticides [27, 28]. Although no bioassays were performed in the present study to evaluate the susceptibility of *An. ziemanni* and *An. pharoensis* to DDT and pyrethroids, previous studies conducted in the area and surrounding localities indicated a low susceptibility of local anopheline species to these insecticides [8, 10, 24]. It should be important for future studies to explore the presence of other resistance mechanisms also in secondary vector species as a recent study indicated the implication of cuticular resistance in *An. pharoensis* samples resistant to DDT [10].

## Conclusions

The spread of *kdr* alleles in other anopheline species is problematic for the use of pyrethroids in public health. Even though, during the last distribution campaign new-generation bed nets, pyrethroid-piperonyl butoxide (PBO) (Olyset Plus^®^), and interceptor^®^ G2 (IG2), combining pyrethroids with other active ingredients were distributed to combat resistant vector populations [1], the impact of this new control strategy is still awaited. The rapid expansion of resistance in vector populations must therefore continue to be the subject of particular attention, as it could compromise the control efforts implemented in the field.

## Data Availability

No datasets were generated or analyzed during the current study.
